# Defining Fulminant *Clostridioides difficile* Infections: Assessing the Utility of Hypotension as a Diagnostic Criterion

**DOI:** 10.1093/ofid/ofaf033

**Published:** 2025-01-22

**Authors:** Hubert C Chua, Taryn A Eubank, Allen Lee, Krishna Rao, Jinhee Jo, Kevin W Garey, Anne J Gonzales-Luna

**Affiliations:** Division of Pharmacy Practice, Arnold and Marie Schwartz College of Pharmacy, Long Island University, Brooklyn, New York, USA; Department of Pharmacy Practice and Translational Research, University of Houston College of Pharmacy, Houston, Texas, USA; Division of Gastroenterology and Hepatology, University of Michigan, Ann Arbor, Michigan, USA; Division of Infectious Diseases, University of Michigan, Ann Arbor, Michigan, USA; Department of Pharmacy Practice and Translational Research, University of Houston College of Pharmacy, Houston, Texas, USA; Department of Pharmacy Practice and Translational Research, University of Houston College of Pharmacy, Houston, Texas, USA; Department of Pharmacy Practice and Translational Research, University of Houston College of Pharmacy, Houston, Texas, USA

**Keywords:** area under the receiver operating characteristic curve, CDI severity criteria, mean arterial pressure, systolic blood pressure, vasopressor

## Abstract

**Background:**

Fulminant *Clostridioides difficile* infection (FCDI) is associated with a 30%–40% mortality rate. Guideline definitions for FCDI severity classification include ileus, megacolon, shock, or hypotension. However, no hypotension definition is provided, making application of the definition challenging. The objective of this study was to assess optimal hypotension definitions for FCDI severity criteria.

**Methods:**

This was a multicenter cohort study involving 1172 hospitalized patients diagnosed with *C difficile* infection (CDI) from 2015 to 2022 (Houston cohort). Patients were assessed for a composite endpoint of colectomy or mortality within 30 days of diagnosis. The ability of the CDI severity criteria to predict the composite endpoint was assessed using 2 definitions of hypotension (systolic blood pressure [SBP] ≤90 mm Hg and mean arterial pressure [MAP] ≤65 mm Hg) through multivariable regression models. A separate CDI cohort of 494 hospitalized patients validated the results (Midwest cohort).

**Results:**

The composite endpoint was similar in the Houston cohort (98 patients [8.4%]) and the Midwest cohort (26 patients [5.3%]). Using either a MAP ≤65 mm Hg or SPB ≤90 mm Hg as criteria for hypotension was the best-performing model in both the development and validation cohorts. Removal of hypotension was the worst-performing model in both cohorts.

**Conclusions:**

Inclusion of hypotension, defined as SBP ≤90 mm Hg or MAP ≤65 mm Hg, was an important component of FCDI severity criteria, significantly improving the predictive ability to identify FCDI patients at risk for poor outcomes.

## Teaser

Defining fulminant *Clostridioides difficile* infection remains challenging due to the lack of hypotension definition. Analysis supports that defining hypotension as systolic blood pressure ≤90 mm Hg or mean arterial pressure ≤65 mm Hg best correlates with worse outcomes.

*Clostridioides difficile* infection (CDI) is associated with increased mortality, complications post-CDI, and economic burden to patients and healthcare facilities [1–3]. Clinical presentation of CDI patients is heterogenous, with symptoms ranging from asymptomatic colonization or mild diarrhea to toxic megacolon, which often leads to death [4, 5]. Various risk factors including host, microbial, gut microbiome, or a combination of all these have been implicated in increasing the risk of CDI severity [6–11]. Due to this, updated guidelines established by the Infectious Diseases Society of America (IDSA) and the Society for Healthcare Epidemiology of America (SHEA) in 2017 endorsed the classification of CDI into nonsevere, severe, and fulminant CDI (FCDI) [12].

FCDI is associated with a 30%–40% mortality rate and prolonged admission in the intensive care unit (ICU) setting [6]. Criteria of FCDI classification based on the 2017 IDSA/SHEA CDI guidelines includes the presence of ileus, toxic megacolon, shock, or hypotension. No definition for hypotension is provided in the 2017 IDSA/SHEA guidelines, making application heterogenous and challenging. Many definitions for hypotension exist, but no study has explicitly identified an objective criterion that best correlates with worse outcomes associated with FCDI [13]. The objective of this study was to assess different hypotension definitions as a FCDI severity criterion.

## METHODS

### Study Design

This was a multicenter cohort study involving CDI patients aged ≥18 years who received care at 2 hospital systems (consisting of 12 institutions) in Houston, Texas, from July 2015 to February 2022. Results from the Houston cohort were validated using a cohort of patients aged ≥18 years diagnosed with CDI at the University of Michigan, the University of Wisconsin, and the University of Chicago from 2010 to 2016 (Midwest cohort), as previously described [14]. Patients with unexplained new-onset diarrhea (defined as ≥3 unformed stools in 24 hours) were tested for CDI at the discretion of the primary medical team. *Clostridioides difficile* diagnostic testing was performed using either a nucleic acid amplification test or toxin enzyme immunoassay. Patients with missing clinical and laboratory data necessary for CDI severity classification and outcomes analysis were excluded. In patients with multiple CDI episodes, only the first encounter during the study period meeting the inclusion criteria was assessed. This study was approved by the University of Houston Committee for the Protection of Human Subjects and the institutional review board at University of Michigan Midwest with a waiver of informed consent. Definitions for both recurrent CDI and healthcare facility–onset CDI (HO-CDI) cases were based on the multidrug-resistant organism/CDI module published by the Centers for Disease Control and Prevention's National Healthcare Safety Network [15].

### Primary Outcome, Definitions, and Severity Classification

The primary outcome was a composite of colectomy or death attributable to CDI within 30 days of stool collection. Medical records were reviewed retrospectively for patient demographics, clinical characteristics, and outcomes data through each hospital institution's electronic health record system. Baseline characteristics, comorbidities, and the Charlson Comorbidity Index (CCI) score were assessed on the date of admission or first encounter [16]. Laboratory parameters recorded within 24 hours of stool collection date (CDI diagnosis date) included white blood cell (WBC) count and serum creatinine (SCr). Similarly, vital signs, such as systolic blood pressure (SBP), diastolic blood pressure (DBP), and mean arterial pressure (MAP), were recorded within 24 hours of CDI diagnosis. If multiple measurements were available, the lowest value was used. MAP was calculated using the formula MAP = DBP + (1/3 * [SBP – DBP]). Vasopressor utilization was defined as the administration of norepinephrine, epinephrine, vasopressin, or dopamine infusions 48 hours following CDI diagnosis. Any hospital admission requiring an overnight stay within 12 weeks of CDI diagnosis was also recorded.

CDI severity was based on clinical definitions published by the 2017 IDSA/SHEA CDI guidelines [12]. In the Houston cohort, all patients on vasopressors or with CDI-associated ileus or toxic megacolon were categorized as FCDI. In the Midwest cohort, all patients in the ICU and mechanically ventilated on the day of CDI stool collection test were categorized as FCDI. Dummy variables for FCDI hypotension severity definitions were then added to the base FCDI definitions using a requirement of SBP ≤90 mm Hg or MAP ≤65 mm Hg or both as hypotension definitions.

### Statistical Analysis

Characteristics of the patient cohorts were analyzed using descriptive statistics. Test for normality was performed using the Shapiro-Wilk test. Continuous and categorical data were reported as mean ± standard deviation, median with interquartile range, or frequency with percentage. Comparisons between patient groups were performed using Mann-Whitney *U* test or Student *t* test for continuous variables, and χ^2^ test or Fisher exact test for categorical variables, as appropriate.

Multivariable logistic regression models were built to assess the effect of different hypotension definitions on the likelihood of the composite outcome. In the Houston cohort, the base case analysis consisted of patients requiring vasopressors or with ileus or toxic megacolon. Three additional models were built to assess the inclusion of SBP ≤90 mm Hg, MAP ≤65 mm Hg, or both as hypotension definitions. For each model, the remaining non-FCDI patients were then categorized to nonsevere or severe CDI classifications based on WBC and SCr.

In the Midwest validation cohort, the base case analysis consisted of patients with FCDI admitted to the ICU and mechanically ventilated. Identical to the Houston cohort, 3 additional models were built to assess the inclusion of SBP ≤90 mm Hg, MAP ≤65 mm Hg, or both as hypotension definitions. For each model, the remaining non-FCDI patients were then categorized to nonsevere or severe CDI classifications based on WBC and SCr according to the definitions established by the 2017 IDSA/SHEA CDI guidelines. Each CDI severity model's ability to predict colectomy or death within 30 days of CDI diagnosis was compared using the area under the receiver operating characteristic curve (AUROC), sensitivity, specificity, and positive/negative predictive values. Sensitivity analyses utilizing classification and regression tree (CART) analysis was performed to identify the SBP threshold that best predicts the primary composite outcome. Subsequently, CDI severity models within 5 mm Hg and 10 mm Hg of the CART-derived cutoff value were created, and their predictive performances were compared to the original definitions prespecified prior to starting the study. All data analysis was performed using R version 4.2.0 software (R Foundation for Statistical Computing, Vienna, Austria). The main packages used in this study were *ggplot2*, *pROC*, and *rpart*.

## RESULTS

### Patient Characteristics

**Figure 1.**
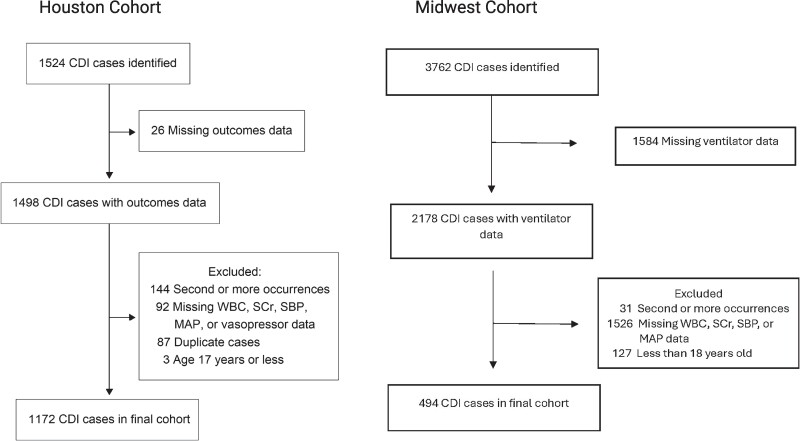
Study flow diagram for the Houston and Midwest study cohorts. Abbreviations: CDI, *Clostridioides difficile* infection; MAP, mean arterial pressure; SBP, systolic blood pressure; SCr, serum creatinine; WBC, white blood cell count.

A total of 1524 CDI cases were identified from the Houston cohort, with exclusions due to recurrent CDI episode (n = 144), missing WBC, SCr, SBP, MAP, or vasopressor use data (n = 92), non-CDI-related readmissions (n = 87), missing outcomes data (n = 26), or <18 years of age (n = 3). The final Houston study cohort consisted of 1172 CDI patients (Figure 1). Characteristics of the study cohort are summarized in Table 1. Ninety-eight (8.4%) patients experienced the primary composite outcome. These patients were older (73 years vs 66 years), were of non-White descent (53.1% vs 57.6%), had a higher median CCI score (3 vs 2), had a longer median admission stay (9 days vs 7 days), and had a higher likelihood of HO-CDI.

**Table 1.** Characteristics of the Study Cohort

Variable	Houston Cohort			Midwest Cohort		
	Colectomy or Death Within 30 d of CDI Diagnosis		*P* Value	Colectomy or Death Within 30 d of CDI Diagnosis		*P* Value
	No (n = 1074)	Yes (n = 98)		No (n = 468)	Yes (n = 26)	
Age, y, median (IQR)	66.0 (53.0–76.0)	72.5 (62.0–82.8)	<.001	60.0 (47.0–70.0)	58.5 (53.3–66.3)	.948
Sex, female	615 (57.3)	51 (52.0)	.372	226 (48.3)	12 (46.2)	.992
Race/ethnicity			.035	…	…	.707
White, non-Hispanic	619 (57.6)	52 (53.1)		397 (84.8)	23 (88.5)	
African American, non-Hispanic	217 (20.2)	23 (23.5)		32 (6.8)	1 (3.8)	
Hispanic	184 (17.1)	16 (16.3)		14 (3.0)	0 (0.0)	
Asian	17 (1.6)	2 (2.0)		NA	NA	
Other/not reported	37 (3.4)	5 (5.1)		25 (5.3)	2 (7.7)	
Hospital organization			.698	…	…	
Hospital 1	112 (10.4)	12 (12.2)		…	…	
Hospital 2	962 (89.6)	86 (87.8)		…	…	
CCI score, median (IQR)	2 (1.0–4.0)	3 (2.0–6.0)	<.001	2 (1.0–3.0)	3 (1.0–4.0)	.025
Length of stay, d, median (IQR)	7 (4.0–13.0)	9 (5.0–17.8)	.008	…	…	
Admitted in the last 12 wk^a^	582 (54.2)	65 (66.3)	.065	…	…	
HO-CDI	508 (47.3)	60 (61.2)	.008	…	…	
WBC count, cells/µL, median (IQR)	10.3 (6.9–15.2)	14.4 (10.0–22.7)	<.001	10.8 (7.5–15.3)	22.9 (9.1–34.5)	<.001
SCr, mg/dL, median (IQR)	1.0 (0.7–1.9)	1.7 (0.9–3.1)	<.001	0.9 (0.7–1.3)	1.8 (1.1–2.7)	<.001
SBP, mm Hg, median (IQR)	107 (96.0–118.0)	92 (80.0–105.0)	<.001	92 (81.0–105.0)	75 (67.5–84.8)	<.001
MAP, mm Hg, median (IQR)	73.5 (66.0–82.0)	63.5 (56.3–75.0)	<.001	68.5 (61.0–78.0)	54.5 (47.5–60.3)	<.001
Vasopressor	55 (5.1)	33 (33.7)	<.001	…	…	
Mechanical ventilation in the ICU	…	…		26 (5.6)	8 (30.8)	<.001

Data are presented as No. (%) unless otherwise indicated.

Abbreviations: CCI, Charlson Comorbidity Index; CDI, *Clostridioides difficile* infection; HO-CDI, healthcare facility–onset *Clostridioides difficile* infection; ICU, intensive care unit; IQR, interquartile range; MAP, mean arterial pressure; NA, not available; SBP, systolic blood pressure; SCr, serum creatinine; WBC, white blood cell.

^a^Excludes 2 missing data in entire cohort.

In the Midwest cohort, a total of 3762 CDI cases were identified, with exclusions due to missing ventilator status (n = 1584), missing WBC, SCr, SBP, or MAP values (n = 1526), recurrent CDI episode (n = 31), or <18 years of age (n = 127). The final Midwest study cohort consisted of 494 CDI patients (Figure 1). Twenty-six (5.3%) patients experienced the primary composite outcome. In contrast to the Houston cohort, the demographics (age, sex, and race) of these patients were not significantly different compared to those who did not have a CDI-attributable colectomy or death within 30 days of CDI diagnosis (Table 1).

### Primary Outcome Analysis

**Figure 2.**
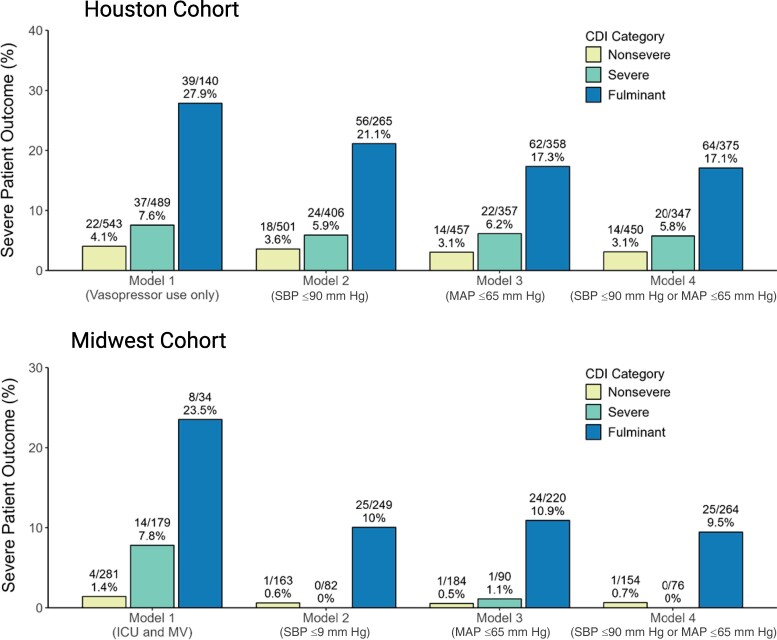
Comparison of patients who experienced colectomy or death within 30 days of *Clostridioides difficile* infection (CDI) diagnosis based on CDI categories with varying hypotension criteria in the Houston and Midwest study cohorts. Model 1 is the base case analysis, with each subsequent model adding a hypotension variable. Abbreviations: CDI, *Clostridioides difficile* infection; ICU, intensive care unit; MAP, mean arterial pressure; MV, mechanical ventilation; SBP, systolic blood pressure.

**Figure 3.**
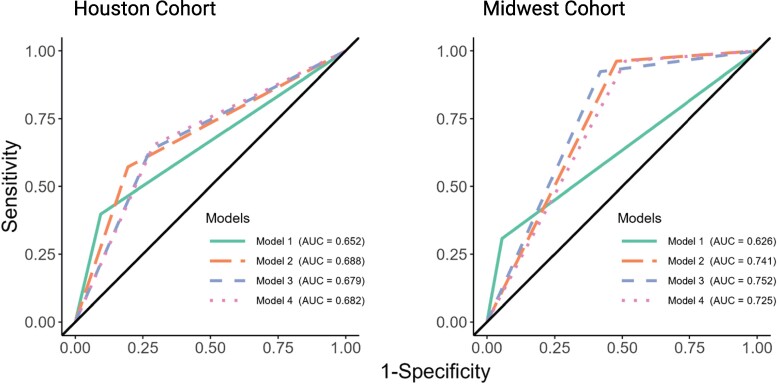
Receiver operating characteristic with area under the curve (AUC) for different fulminant *Clostridioides difficile* models.

In the Houston cohort, 4 patients had toxic megacolon (0.34%), 59 had ileus (5%), and 88 had vasopressor use (7.5%) resulting in a base FCDI rate of 12% (n = 140). Rates of colectomy or death within 30 days of CDI diagnosis were consistently higher in patients with FCDI in the base model and using the varying hypotension definitions (range, 17.1%–27.9%) compared to severe (range, 5.8%–7.6%) or nonsevere (range, 3.0%–4.1%) CDI (Figure 2). In the Midwest cohort, 34 patients were in the ICU and mechanically ventilated at the time of stool collection, resulting in a base FCDI rate of 6.9%. Rates of colectomy or death within 30 days of CDI diagnosis were also consistently higher in patients with FCDI in the base model and using the varying hypotension definitions (range, 9.5%–23.5%) compared to severe (range, 0–7.8%) or nonsevere (range, 0–1.4%) CDI (Figure 2). Values for the AUROC and model performance characteristics measures are shown in Figure 3 and Table 2. Baseline models without incorporation of hypotension definitions consistently performed the worst in both cohorts (area under the curve [AUC], 0.626–0.652). All models significantly improved upon the base model in the Houston (AUC, 0.679–0.688) and Midwest (AUC, 0.725–0.752) cohorts with SBP ≤90 mm Hg performing best in the Houston cohort and MAP ≤65 mm Hg performing best in the Midwest cohort. The performance characteristics of each model in the Houston and Midwest cohorts are shown in Table 2. All models achieved a negative predictive value of >90%.

**Table 2.** Performance Characteristic Measures of Fulminant *Clostridioides difficile* Infection Based on Varying Hypotension Criteria

Fulminant CDI Model	Sensitivity	Specificity	PPV, %	NPV, %	AUROC
Houston cohort^a^					
Base case analysis (vasopressor use, ileus, or toxic megacolon)	0.40	0.91	27.9	94.3	0.652
Model 2: SBP ≤90 mm Hg	0.57	0.81	21.1	95.4	0.688
Model 3: MAP ≤65 mm Hg	0.63	0.72	17.3	95.6	0.679
Model 4: SBP ≤90 mm Hg or MAP ≤65 mm Hg	0.65	0.71	17.0	95.7	0.682
Midwest cohort					
Base case analysis (ICU and MV)	0.31	0.94	23.5	96.1	0.626
Model 2: SBP ≤90 mm Hg	0.96	0.52	10.0	99.6	0.741
Model 3: MAP ≤65 mm Hg	0.92	0.58	10.9	99.3	0.752
Model 4: SBP ≤90 mm Hg or MAP ≤65 mm Hg	0.96	0.49	9.5	99.6	0.725

Abbreviations: AUROC, area under the receiving operator characteristic curve; CDI, *Clostridioides difficile* infection; ICU, intensive care unit; MAP, mean arterial pressure; MV, mechanical ventilation; NPV, negative predictive value; PPV, positive predictive value; SBP, systolic blood pressure.

^a^In all models, no cases had ileus and toxic megacolon simultaneously.

### Sensitivity Analysis

Using the Houston cohort, CART analysis determined that colectomy or death within 30 days of CDI diagnosis was most significantly correlated with SBP <85 mm Hg (34.6% experienced the primary endpoint with SBP <85 mm Hg vs 5.8% with SBP ≥85 mm Hg). Sensitivity models were built on SBP by varying the definition of hypotension by 90 ± 5 mm Hg and 90 ± 10 mm Hg and comparing composite outcomes and AUROC results based on results from the sensitivity analysis. Results of the sensitivity analysis were similar to those of the primary analysis (Supplementary Figure 1), with the prespecified SBP definition having the highest AUROC (Supplementary Figure 2).

## DISCUSSION

CDI has a large spectrum of presentation and varying poor outcomes from persistent diarrhea, ICU admissions, colectomy, and death. Given the wide spectrum of disease presentation and multiple poor outcomes, existing CDI severity classification models are heterogenous in their model variables and outcomes measured [17]. The most commonly used CDI severity measure is the IDSA/SHEA guideline definitions [18]. Studies that have previously assessed the IDSA/SHEA CDI severity criteria to predicting outcomes in patients with CDI have resulted in AUROCs that ranged from 0.57 to 0.74 [17–19]. One source of this variability is likely the clinical criteria used to define hypotension in patients with severe/fulminant CDI. A systematic literature review identified varying definitions of hypotension such as an absolute SBP threshold (<80 to <100 mm Hg) or a relative decrease from baseline (>10% to >40% from baseline) [13]. Although many definitions have been proposed, the Society of Critical Care Medicine’s Surviving Sepsis Campaign defines hypotension as a MAP ≤65 mm Hg while others use an SPB ≤90 mm Hg [20–22]. These values were also the preferred definitions for hypotension in a survey of >1200 ICU providers [23]. For these reasons, we chose to evaluate MAP ≤65 mm Hg and SPB ≤90 mm Hg as defining variables for hypotension to be used in FCDI severity criteria. Strengths of this study include its multicenter study design; standardized definitions of demographic, clinical, and laboratory variables; and strict inclusion of patients with complete data. Results of the study demonstrated that using either a MAP ≤65 mm Hg or SPB ≤90 mm Hg as criteria for hypotension was the best-performing criteria in both our development and validation cohorts. We propose that these hypotension definitions be included as CDI severity classification criteria in future IDSA/SHEA CDI guidelines.

This study has certain limitations. We purposely chose CDI-attributable mortality and colectomy for this study to limit bias associated with outcomes evaluations. Many other important CDI-related outcomes exist, including ICU admission; these will need to be evaluated in the future. Two measures of hypotension were evaluated: the absolute lowest SBP or MAP value within 24 hours from stool collection. Other methods to define hypotension exist, including relative change compared to a baseline measurement. Similar to creatine measurements, we chose absolute values as the ability to measure baseline values may not be present for all cases [24]. Blood pressure readings are measured numerous times per day in hospitalized patients, thus being readily available data for severity classification. More advanced calculations over time could also be considered but would increase the complexity of the severity definitions. The Midwest validation dataset did not contain a variable for vasopressors. For this reason, we chose the close proxy of ICU admission with mechanical ventilation to represent a similar patient population. The validation cohort collected data from 2010 to 2016; further validation from contemporary datasets would further validate these findings.

In conclusion, using a hypotension definition of either SBP ≤90 mm Hg or MAP ≤65 mm Hg to define FCDI best correlated with colectomy or death within 30 days of CDI diagnosis. These definitions of hypotension should be included in future FCDI severity criteria including future updates to the IDSA/SHEA CDI guidelines.

## Supplementary Material

ofaf033_Supplementary_Data

## Supplementary Data

Supplementary materials are available at *Open Forum Infectious Diseases* online. Consisting of data provided by the authors to benefit the reader, the posted materials are not copyedited and are the sole responsibility of the authors, so questions or comments should be addressed to the corresponding author.
